# Pharmacist-led deprescribing of cardiovascular and diabetes medication within a clinical medication review: the LeMON study (Less Medicines in Older Patients in the Netherlands), a cluster randomized controlled trial

**DOI:** 10.1007/s11096-025-01863-w

**Published:** 2025-01-23

**Authors:** Jamila Abou, Petra J. M. Elders, Danielle Huijts, Rob van Marum, Jacqueline Hugtenburg

**Affiliations:** 1 Department of Clinical Pharmacology and Pharmacy, Amsterdam University Medical Centers, Location VUMC, Amsterdam, The Netherlands; 2 Department of General Practice, Amsterdam UMC, Location VU, Amsterdam Public Health Research Institute, Amsterdam, The Netherlands; 3 Department of Elderly Care Medicine, Amsterdam Public Health Research Institute, Amsterdam UMC (Location VUmc), Amsterdam, The Netherlands; 4 Department of Clinical Pharmacology, Jeroen Bosch Hospital, ’s-Hertogenbosch, The Netherlands

**Keywords:** Frail elderly, Medication reconciliation, Medication review, Pharmacists, Polypharmacy

## Abstract

**Background:**

Deprescribing inappropriate cardiovascular and antidiabetic medication has been shown to be feasible and safe. Healthcare providers often perceive the deprescribing of cardiovascular and antidiabetic medication as a challenge and therefore it is still not widely implemented in daily practice.

**Aim:**

The aim was to assess whether training focused on conducting a deprescribing-oriented clinical medication review (CMR) results in a reduction of the inappropriate use of cardiovascular and antidiabetic medicines.

**Method:**

A cluster randomized controlled trial involving 20 community pharmacists, who conducted a clinical medication review in 10 patients. The intervention group received training on deprescribing. Patients 70 years or older with polypharmacy having a systolic blood pressure below 140 mmHg and using antihypertensive medication and/or an HbA1c level below 54 mmol/mol and using antidiabetic medication, were included. Follow-up took place within 4 weeks (T1) and after 3 months (T2). The primary outcome measure was the proportion of patients with one or more cardiovascular and antidiabetic medicine deprescribed within 3 months after the CMR (T2).

**Results:**

A total of 71 patients in the intervention group and 69 patients in the control group were included. At T2, 32% of patients in the intervention group and 26% in the control group (OR 1.4, CI 0.65–2.82, *p* = 0.413) had one or more cardiovascular or antidiabetic medicines discontinued. Regarding any medication, these percentages were 51% and 36%, (OR 1.8, CI 0.92–3.56, *p* = 0.085) respectively.

**Conclusion:**

Increased awareness and ability of community pharmacists to deprescribe medication and use of general practitioners’ data, led community pharmacists and general practitioners to successfully conduct a more deprescribing-focused CMR in daily practice. Further research is needed to assess the necessity of additional training to optimize the deprescribing of cardiovascular and antidiabetic medication.

The study was registered at The Netherlands Trial Register (registration no: NL8082).

**Supplementary Information:**

The online version contains supplementary material available at 10.1007/s11096-025-01863-w.

## Impact statements

- The use of medication records from community pharmacists and clinical data from general practitioners could improve the targeting of patients who may benefit from deprescribing antihypertensive and antidiabetic medication.
- The implementation of deprescribing of antihypertensive and antidiabetic medication within the context of clinical medication review needs further study.
- Effectively conducting deprescribing-focused clinical medication review requires both training of community pharmacists and adequate communication and collaboration between community pharmacists and general practitioners.

## Introduction

Polypharmacy is associated with medication-related hospital admissions [1]. Especially in older patients, polypharmacy can lead to an increased risk of drug-related problems. Due to age-related changes in pharmacodynamics and pharmacokinetics and increased frailty, potential harm can outweigh the clinical benefits of preventive medication over time. Preventive medication initiated in the past, can therefore become inappropriate, and its use may result in increased morbidity, hospitalizations and health care costs [1–5]. This indicates the need to evaluate prescriptions for preventive medication in older persons, especially frail ones. Deprescribing, defined as the process of withdrawing inappropriate medication under the supervision of a healthcare provider (HCP), aims to manage polypharmacy and improve patient outcomes [6]. It may serve as an important pharmacotherapeutic tool to address inappropriate polypharmacy in frail older patients with multimorbidity. This process can optimize medication use in terms of both safety and outcomes, potentially enhancing patients' quality of life [7, 8]. Conducted at regular intervals, a clinical medication review (CMR) is performed in primary care and nursing home care to manage polypharmacy in older patients with multimorbidity to prevent drug-related problems leading to falls, hospital admissions, a lower quality of life, increased mortality and excessive health care costs [5, 9–23].

Although evidence is not strong and clear, deprescribing of inappropriate (preventive) cardiovascular and antidiabetic medication seems feasible and safe [9, 24–32]. Therefore, incentives for deprescribing this medication have been introduced in guidelines for the treatment of cardiovascular diseases and type 2 diabetes mellitus (T2DM) [25–28, 32–36]. HCPs, however, often experience the deprescribing of cardiovascular and antidiabetic medication as a challenge. As the result, it is still not widely implemented in daily practice [9, 14, 37–41]. Barriers include a lack of evidence that discontinuation is more beneficial than continuation, HCP and patient uncertainty, fear of negative consequences, reluctance among patients or their relatives to accept changes to medication, a lack of knowledge, time, training or support to allow HCPs to effectively perform deprescribing and suboptimal HCP collaboration [9, 13, 14, 37–44]. In the Netherlands community pharmacists (CPs) are responsible for offering pharmacotherapeutic care and thereby play a significant role in medication management in primary care [11–13, 21, 22]. CPs have also been tasked with conducting CMRs annually in older patients with polypharmacy in collaboration with general practitioners (GPs), geriatricians, specialists geriatric medicine and other HCPs [12–14, 16, 18, 44–46]. Recommendations on how to perform a patient-centered CMR, pharmacotherapeutic criteria and tools are provided by the Multidisciplinary Guideline ‘Polypharmacy in older patients’ [44–46]. Despite its mandatory nature and remuneration, CMR implementation in practice is still not without problems, especially with regard to the deprescribing of (preventive) cardiovascular and antidiabetic medication [13–16, 37, 38, 40, 44–47]. As a remedy, CMR eligibility has been restricted to high-risk patients and recommendations on deprescribing have been added [44, 46]. Criteria and considerations have now systematically been summarized to facilitate patient-centered decision-making to continue, adjust or discontinue medication. However, to achieve full implementation of the Multidisciplinary Guideline ‘Polypharmacy in older patients’ in daily practice, the CPs, GPs and other HCPs involved need training and support, especially with regard to patient selection, decision-making and HCP collaboration/patient communication [8, 9, 12–15, 40, 45–50].

### Aim

The aim of this study was to assess whether training for CPs on how to conduct a more deprescribing-focused CMR, would result in a greater reduction in the inappropriate use of (preventive) cardiovascular and oral antidiabetic medication in terms of discontinuation, as compared to CMRs conducted without prior training.

### Ethics approval

The study protocol was approved by the Medical Ethics Committee of the VU University Medical Center in Amsterdam (2019.326).

## Method

### Study design and setting

The ‘Less Medicines in Older Patients in the Netherlands’ (LeMON) study, is a cluster randomized controlled trial conducted between November 2020 and December 2021. The study evaluated the effects of training for CPs aimed to conduct a more deprescribing-focused CMR with regard to cardiovascular and oral antidiabetic medication in daily practice.

The BENU pharmacy chain is the largest pharmacy chain in the Netherlands, and the pharmacists generally follow the same procedures in patient care. The BENU pharmacy chain provided a longlist of eligible CPs working in medium-sized and large BENU pharmacies across the country. In the Netherlands, a medium-sized pharmacy serves between 5000 and 10,000 patients. A large pharmacy serves between 15,000 and 20,000 patients.

Thirty-five CPs were invited to participate, 20 of these participated in the study and were block-randomized in Castor EDC, a web-based data management platform [51]. CPs randomized to the intervention group were asked to participate in a deprescribing training. To minimize performance bias CPs in the control group were not informed about the content of the training.

**Fig. 1:**
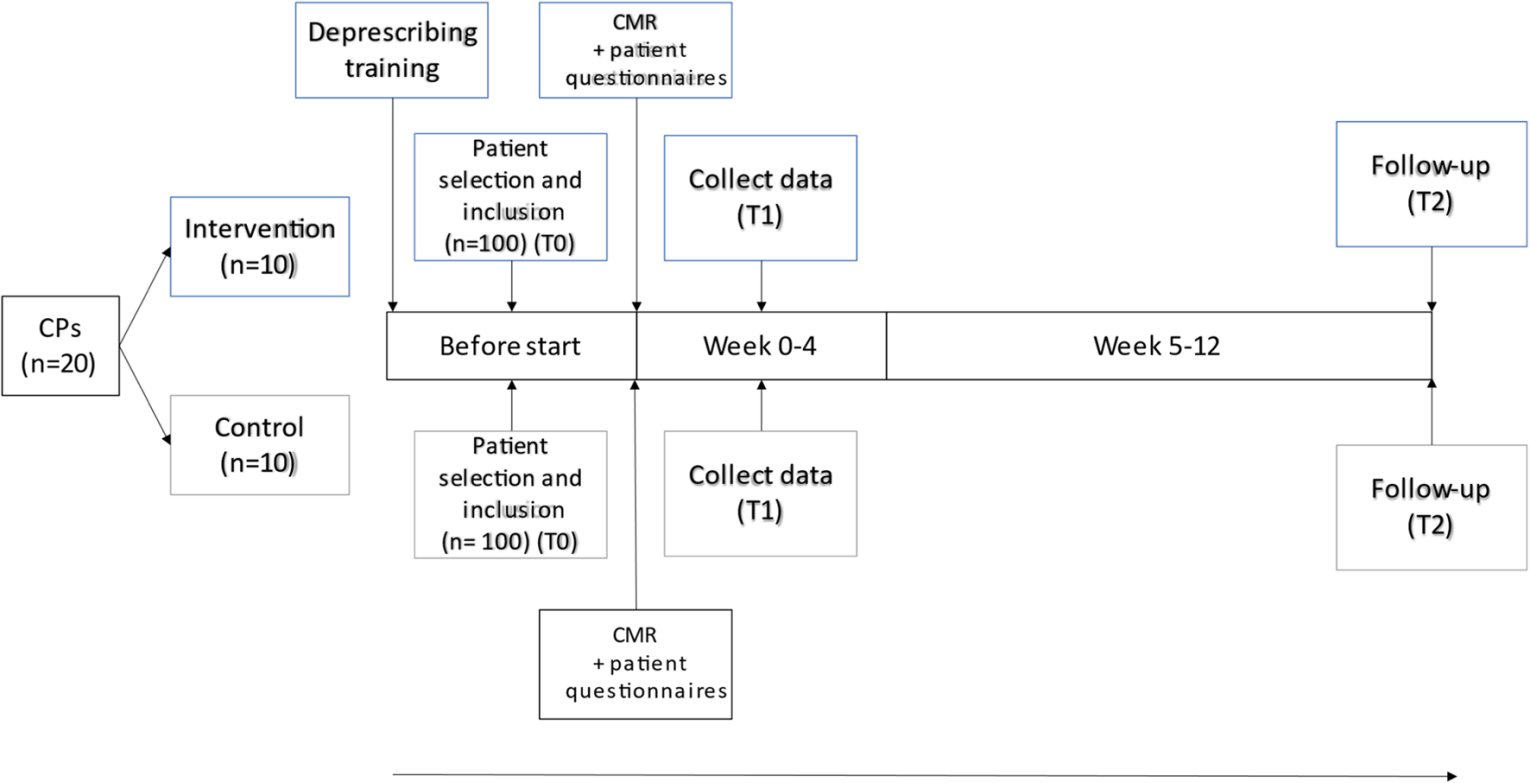
Flowchart of the outline the study design. Abbreviations: CP = community pharmacist, CMR = clinical medication review

CMRs and follow-up meetings with patients were in the pharmacies or completed electronically or by telephone, depending on the CPs’ own working procedures and CP and patient preferences. Outcomes were reported within 4 weeks after each CMR (T1) and after 3 months (T2). A flowchart of the study design is presented in Fig. 1.

### LeMON intervention

LeMON is part of a collaboration called DISCARDT, in which three research groups in the Netherlands have received funding from The Royal Dutch Pharmacists Association (KNMP) to develop tools and strategies aimed at reducing the medication burden in individuals with T2DM and/or cardiovascular diseases. The training included the following key elements: 1. knowledge on the deprescribing of cardiovascular medication, 2. identification of eligible patients, 3. addressing barriers and facilitators, 4. applying shared decision-making and 5. monitoring of CMRs. More detailed information about the training can be found in the supplementary material.

### Patient selection

Patients were selected by the CPs via data from the Pharmacy Information and Administration System (PIAS) and the General Practice Information and Administration System (GIAS). This was done in the same manner for both the control and intervention groups. The inclusion criteria for patients were as follows: 70 years or older and chronic use of five or more medicines including at least one antihypertensive or antidiabetic medicine (Anatomical Therapeutic Chemical [ATC] classification codes: A10: diabetes and C: cardiovascular system). In addition, patients should have had a recently measured (< 6 months before inclusion) systolic blood pressure < 140 mmHg and/or a HbA1c level < 54 mmol/mol. Life expectancy should be more than 3 months as estimated by the GP.

The CPs selected eligible patients from their PIAS and contacted the GPs of these patients for collaboration and GIAS information on additional inclusion and exclusion criteria. In the case of linked systems (Pharmacom®-Medicom®) CPs had direct access to these data. CPs invited eligible patients by phone or at pharmacies. They were subsequently informed about the study by their CP and were asked to provide informed consent before participation.

### Sample size calculation

On the basis of previous studies it was assumed that in 40% of the patients the number of cardiovascular and antidiabetic medicines could be reduced [52]. With an α of 5% and power of 90%, 80 patients were needed in each arm. Since the CPs work for the same pharmacy chain, BENU Apotheken, they are trained in the provision of pharmaceutical care and conducting CMRs in a similar manner. Considering 10% loss to follow up, a total of 100 patients would have to be recruited per arm. To keep the workload per CP as low as possible, in view of the estimated number of patients fulfilling the inclusion criteria and a recruitment period of 6 months, conducting a CMR in 10 patients per CP was considered feasible.

### Primary outcome measures

The primary outcome measure was the proportion of patients with one or more cardiovascular and antidiabetic medicine deprescribed within 3 months after the CMR (T2). Deprescribing was defined as stopping medication, reducing the dose of medication or substituting a medicine for a less potent alternative.

### Secondary outcome measures

The secondary outcome measures were the type of medication and the number of proposed deprescribing interventions at the follow-up meetings with GPs and patients 1–4 weeks after the CMR (T1).

### Data analysis

All analyses were performed via the Statistical Package for the Social Sciences version 15 software (SPSS). Descriptive statistics included the mean and standard deviation for normally distributed variables and median and interquartile range for nonparametric variables. Study groups were compared via the χ2 or Fisher’s exact test for categorical variables and the Student’s t-test for normally distributed continuous variables. Following descriptive analysis, the data were analysed with a logistic mixed model, which included a random effect model for CPs. Multilevel analysis was used to analyse the effect of clustering patients at the CP level. Age, systolic blood pressure and sex were analysed as potential confounders. Adjustment was made for the number of medication at baseline level by including it as a fixed effect. For patients with T2DM subgroup analyses were performed. All the data were collected via Castor EDC and SPSS and were discussed within the research team and with an expert in statistics. The CONSORT statement was used to evaluate the design of this trial [53].

## Results

**Fig. 2:**
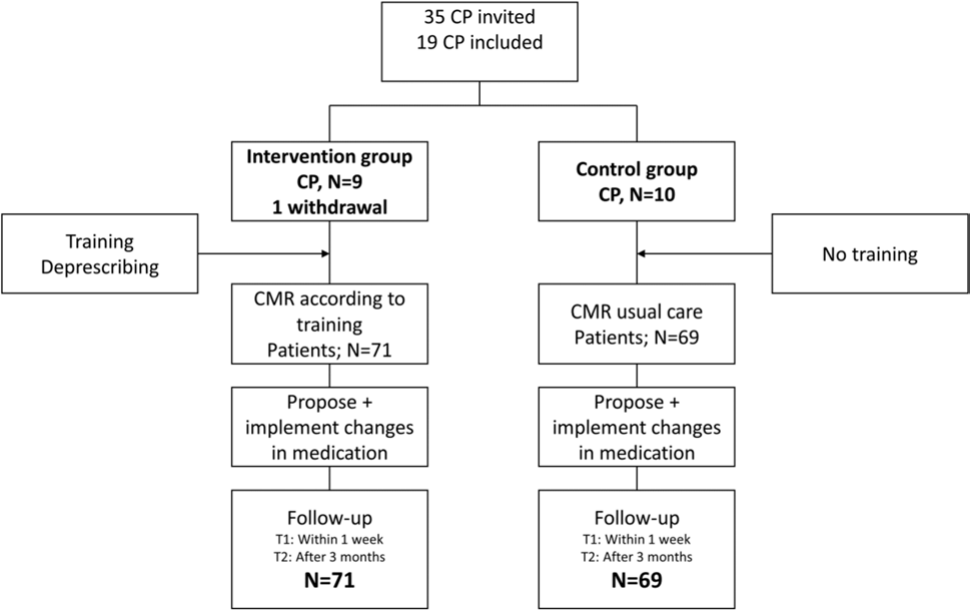
Flowchart of the inclusion and intervention process

An overview of the inclusion of CPs and intervention process is shown in Fig. 2. Between November 2020 and December 2021, 35 BENU pharmacies were invited, of which 19 indicated a willingness to participate and were included. The CPs were randomized to an intervention (N = 9) and control group (N = 10). One CP in the intervention withdrew after randomization (intervention) due to time constraints caused by unexpected staff shortages. The baseline characteristics of the participating CPs are shown in Table 1.

**Table 1:** Baseline characteristics of pharmacists

	Intervention	Control
	n = 8	n = 10
Sex, female, n (%)	8 (100)	7 (70)
Experience as CP, n (%)		
0–5 years	4 (50)	5 (50)
6–15 years	4 (50)	2 (20)
16–25 years		3 (30)
Scale of pharmacy		
Middle-large pharmacy	7	8
Large pharmacy	1	2

The CPs included 140 patients: 71 in the intervention group and 69 in the control group. The baseline characteristics of the patients are shown in Table 2.

**Table 2:** Baseline characteristics of patients at T0

	Intervention	Control	p-value
	n = 71	n = 69	
Age, mean, years (SD)	82 (5)	81 (7)	0.311
Sex, female, n (%)	39 (67%)	34 (50%)	0.098
Systolic blood pressure (mm HG) mean (SD)	129 (12)	131 (11)	0.211
Number of total medication, mean (SD)	11 (3.5)	10 (3)	0.371
Number of cardiovascular and diabetes medication before intervention, mean	3.5	3.6	0.412

Table 3 shows the outcomes of the analysis for the intervention group compared the control group at T1 and T2, with adjustments for the covariates age, sex, blood pressure and number of medication in the regression analysis.

**Table 3:** Univariable and multivariable analyses of primary (T2) and secondary (T1) outcomes, with correction for the covariates age, sex, blood pressure and number of medication

	Intervention n = 71	Control n = 69	Univariable regression Odds ratio (CI)	P-value	Multivariable regression Odds ratio (CI)	P- value
All patients n = 140						
Patients at T1, proposed deprescribing						
Cardiovascular and/or diabetes medication, n (%)	39 (55)	32 (46)	1.41 (0.73–2.74)	0.312	1.33 (0.67–2.64)	0.411
Any medication, n (%)	53 (75)	48 (70)	1.29 (0.61–2.70)	0.503	1.14 (0.52–2.49)	0.752
Patients at T2, actual deprescribing						
Cardiovascular and diabetes medication, n (%)	23 (32)	18 (26)	1.36 (0.65–2.82)	0.413	1.29 (0.59–2.79)	0.519
Any medication, n (%)	36 (51)	25 (36)	1.81 (0.92–3.56)	0.085	1.59 (0.76–3.36)	0.221
Diabetes mellitus type 2 patients n = 65						
Patients at T1, proposed deprescribing						
Cardiovascular and/or diabetes medication, n (%)	16 (52)	14 (45)	1.03 (0.38–2.77)	0.951	1.07 (0.36–3.08)	0.908
Any medication, n (%)	22 (71)	26 (76)	0.56 (0.19–1.65)	0.292	0.55 (0.18–1.70)	0.294
Patients at T2, actual deprescribing						
Cardiovascular and/or diabetes medication, n (%)	12 (39)	7 (21)	1.58 (0.51–4.92)	0.432	1.69 (0.48–5.87)	0.417
Any medication, n (%)	17 (55)	12 (35)	1.72 (0.64–4.65)	0.286	1.71 (0.59–4.99)	0.322

Among the patients who signed an informed consent form at T1, one or more proposals were made for the deprescribing of cardiovascular and antidiabetic medication in 55% and 46% (*p* = 0.411) of the patients in the intervention and control groups, respectively (Table 3). With regard to all medication, these percentages were 75% and 70%, (*p* = 0.752) respectively.

Three months after the CMR (T2) 32% and 26% (*p* = 0.519) of the patients in the intervention and control groups, respectively, discontinued one or more cardiovascular or antidiabetic medicines. With regard to any medication these percentages were 51% and 36%, (*p* = 0.221) respectively. The number of deprescribed medication was highest in the 'antihypertensive' medication group.

In the intervention group two thirds of the CP proposals to deprescribe resulted in the actual deprescribing of both all and cardiovascular or antidiabetic medication (68%, 59%, *p* = 0.221). In the control group this was the case for half of the proposals that were implemented (56%, 52%, *p* = 0.519) (Table 3).

The odds ratio for discontinuing cardiovascular or antidiabetic medication at T2 was 1.4 (CI 0.65–2.28, *p* = 0.413) for the intervention compared to control CPs. The differences with regard to the deprescribing of cardiovascular and antidiabetic medication before and after correction for confounding variables in the regression models were not statistically significant (Table 3).

Table 4 shows the nature and frequency of medication discontinuation in the control and intervention groups. In total in the control and the intervention groups, 28 and 42 medicines were discontinued, respectively.

**Table 4:** Nature and frequency of medicines deprescribed in the intervention and control and groups at T2

	Intervention	Control
Non-CVRM medication deprescribed (ATC), n		
Proton pump inhibitors (A02)	5	2
Medication for benign prostatic hyperplasia (BPH) (G04)	1	1
Pain medication (N02)		1
Inhalation medication (R03)	1	3
Tricyclic antidepressants (N06)	1	1
Antihistamines (R06)	2	
Bisphosphonates (M05)	2	
CVRM medication group deprescribed, ATC, n		
Antiarrhythmic medication (C01)	1	1
Antihypertensives (C02-08)	9	7
Antiplatelet medication (B01)	6	6
Loop diuretics (C03)	4	2
Statins (C10)	6	3
Glucose lowering medication (A10)	4	1

## Discussion

The present study reports the results of a cluster-randomized controlled trial investigating the effects of a training for CPs on the deprescribing of cardiovascular and antidiabetic medication when conducting a CMR in older patients with polypharmacy. For CPs who participated in the training the number of patients in whom (cardiovascular and antidiabetic) medication was deprescribed was greater than for CPs who conducted CMRs without additional training, but the difference was not statistically significant.

In the present study, CPs proposed deprescribing any medication for 75% and 70% of the intervention and control patients, respectively. Proposals for the deprescribing of cardiovascular and antidiabetic medication were made for 55% and 46% of the patients, respectively. Following discussions with GPs and patients, it appeared that after 3 months any medication had actually been deprescribed in 51% and 36% and cardiovascular and antidiabetic medication in 32% and 26% of these patients, respectively. Ultimately, in the intervention group about two thirds and in the control group approximately half of the proposals were actually implemented. The difference was not statistically significant.

In a Canadian CP-led intervention study in older community dwelling patients using medication with a high risk of inducing drug-related problems, oral antidiabetic medication was deprescribed in 30% of the patients who used this medication, for the duration of a 6 months follow-up period [17]. Regardless of the medication used, in the Netherlands follow-up (3 months–1 year) deprescribing rates in CP-led CMR intervention studies in older community dwelling patients ranged from 20 to 50% with those for cardiovascular and antidiabetic medication falling within this range [11, 13, 14, 16]. In studies led by GPs but with CP assistance similar results were obtained [21, 42].

Reviews of both intervention and observational studies on the deprescribing of cardiovascular and antidiabetic medication in different categories of older patients show much wider ranges of deprescribing. For patients with antidiabetic medication with or without a cardiovascular condition rates between 13 and 75% have been reported [27]. A similar wide range (27–94%) was reported in a review on the effects of various deprescribing approaches in older patients with a limited life span or living in nursing homes [32]. However, for community dwelling and nursing home patients with T2DM and low HbA1c levels oral antidiabetic medication deprescribing rates ranging from 14 to 27% were reported whereas for patients with T2DM and a low systolic blood pressure the rates for this medication were between 16 and 19% [29]. A 66% 3- month deprescribing rate of antihypertensive medication was reported in a UK trial in primary care patients with hypertension aged 80 years or older [31]. In this study the largest number of discontinued medicines consisted of antihypertensives. The trigger for modifying or discontinuing medication is likely a clear parameter, such as blood pressure. Low blood pressure may prompt both HCPs and patients to consider discontinuing medication, or perhaps these patients experienced symptoms such as hypotension. Further research is needed to gain a better understanding of the reasons for discontinuing medication.

The results of the present study are somewhat better than those of previous studies evaluating the extent to which conducting a CMR leads to the deprescribing of medication. The full implementation of the Multidisciplinary Guideline ‘Polypharmacy in older patients’, which makes the annual CMR in frail older patients with polypharmacy a mandatory intervention, and the recent addition of guidance on deprescribing are likely to have increased the deprescribing rate over the years [46]. In the present study the difference between the deprescribing rates in the intervention group and the control group was not significant. Most likely this is due to the fact that in the last decade CPs have become aware of deprescribing, both as the result of experience gained in conducting increasing numbers of CMRs and the already wide attention given to deprescribing in Dutch medical and pharmaceutical journals and at local pharmacotherapeutic audit meetings. Moreover, current Dutch guidelines for the treatment of cardiovascular diseases and T2DM, which have been in use for several years, offer a wide variety of options for the tailored treatment of older patients [35, 36]. The deprescribing of medication in the majority of patients in the present study therefore appears to have been primarily due to the ability of CPs to deprescribe cardiovascular and antidiabetic medication.

Proposals for the discontinuation of medication are not always accepted by GPs and other prescribing HCPs, and patients. Proposals to discontinue or adjust medication were made for 70–75% of the patients participating in this study. Although GPs and other HCPs generally recognize the expertise of CPs, several factors may lead GPs and patients to reject CP proposals to discontinue medication [8, 9, 13, 14, 37–41].

### Strengths and weaknesses

A strength of the present study is the use of both CP medication records and GP clinical data in selecting older patients with polypharmacy for a CMR in both the intervention and the control groups. This allowed patients potentially eligible for the deprescribing of cardiovascular and antidiabetic medication to be targeted. Another strength is that deprescribing training and the deprescribing-focused CMR are embedded in the current practice of conducting mandatory annual CMRs by CPs and GPs.

The inclusion period coincided with the COVID-19 pandemic, which made the inclusion process challenging. The initial target for patient inclusion per CP at the onset of the study was 10 patients. However, during the inclusion process, it became apparent that this target was not feasible due to prevailing circumstances, and this number was not achieved by all CPs. Therefore, the non-significant results may also be due to the study being underpowered.

Selection bias may have occurred because intervention CPs could select patients who are more eligible for deprescribing. However, we provided both intervention and control CPs with clear inclusion criteria and a protocol for selecting the patients. Moreover, CPs selected patients within the group of patients for whom a CMR with the GP was already planned. We therefore assume that selection bias is minimal. The GP collaboration required in making CMRs was also increasingly difficult due to a shortage of GPs and the resulting lack of GP time [44]. This also means that there is an obvious need for clear criteria and responsibilities, as well as (electronic) tools that allow the efficient transfer of GP clinical information, facilitate the selection of patients and the acquisition of information from patients and ensure that CMRs including the deprescribing of medication, can be efficiently conducted [8, 9, 12, 18]. In the present study, most CPs had no direct access to blood pressure or HbA1c data. Conducting CMRs and adjusting or deprescribing cardiovascular and antidiabetic medication would be enhanced if PIAS and GIAS were (mandatory) linked so that this information is directly available to CPs. Several studies have indicated that pharmacist-led educational interventions, compared with usual care, resulted in higher rates of discontinuation of cardiovascular and antidiabetic medication [17, 54]. Deprescribing in patients with T2DM has been associated with a reduction in the incidence of hypoglycemia [55]. However, most of these studies were not randomized controlled trials (RCTs), and involved larger sample sizes. Despite this, the evidence regarding the impact of pharmacist-driven interventions in deprescribing remains inconclusive [56, 57]. In the present study the clinical effect of medication discontinuation was also not investigated. Finally, a limitation could be that the effects of deprescribing were assessed only after 3 months, and that there are no longitudinal data.

## Conclusion

A deprescribing training of CPs did not significantly enhance the deprescribing of cardiovascular and antidiabetic medication in older patients with polypharmacy selected on the basis of dispensing data and specific criteria for blood pressure level and HbA1c value. The increased awareness and ability of CPs to deprescribe cardiovascular and antidiabetic medication and use of GP data, led CPs and GPs to successfully conduct a more deprescribing-focused CMR in daily practice. The need for additional CP training and enhanced GP-CP collaboration to optimize the deprescribing of cardiovascular and antidiabetic medication in conducting CMRs requires further development and study.

## Supplementary Information

Below is the link to the electronic supplementary material.

Supplementary file1 (DOCX 27 KB)
