# Supplementary material for: Pharmacist-led deprescribing of cardiovascular and diabetes medication within a clinical medication review: the LeMON study (Less Medicines in Older Patients in the Netherlands), a cluster randomized controlled trial
Source: Int J Clin Pharm. 2025 Jan 23;47(3):717–25. doi: 10.1007/s11096-025-01863-w (PMC12125148; doi:10.1007/s11096-025-01863-w)
Supplement: Supplementary file 1 — Supplementary file1 (DOCX 27 KB) [file 11096_2025_1863_MOESM1_ESM.docx]

Supplementary material : LeMON training

WHAT

The content of the training was based on prior research and addressed the following topics [1] strategies and tools on deprescribing [2] collaboration with other HCPs (multidisciplinary approach), [3] involvement of the patient and patient counselling, [4] composing a deprescribing treatment plan, and [5] monitoring. making.

WHY

The Dutch multidisciplinary guideline module for deprescribing medication was not available and was still under development. There were no deprescribing training. The aim of the training was mainly to broaden the knowledge on deprescribing, addressing barriers and facilitators for deprescribing from the perspective of the patient and HCP and patient-centered decision

HOW

The LeMON training was conducted individually for each pharmacist enrolled in the intervention group. The training was delivered once with a duration of approximately 2.0 hours.

WHERE

Due to COVID-19, the training was conducted individually in an online ZOOM meeting instead of in person in a group meeting.

WHO

The training was developed by JH and PE who are pharmacist and general practitioner with more than 10 years’ experience in providing postgraduate training on optimizing pharmaceutical care.

Overview of the content of the LeMON training:

Abbreviations: CMR: clinical medication review

| **Goals** | **Topic** | **Description of content** | **Method and Materials** |
| --- | --- | --- | --- |
| The objective is to train community pharmacists in competencies regarding conducting CMR, focusing on deprescribing in cardiovascular and diabetes patients | -Knowledge enhancement on deprescribing  -Identify patients eligible for deprescribing cardiovascular and diabetes medication  - Recognize and address barriers and facilitators to deprescribing in patients and HPC  -Apply shared decision making in CMR with focus on deprescribing cardiovascular and diabetes medication | -Guidelines for CMR and research regarding deprescribing  -Case examples with specific recommendations on deprescribing cardiovascular and diabetes medication  -Guidelines on Polypharmacy and patient cases  -Communication skills on patient’s preferences and attitude on deprescribing | Oral presentation, case examples  Oral presentation,  Patient cases  Oral presentation,  Patient cases  Oral presentation and discussion |
|  |  |  |  |
